# Inhibition of GSK 3β Activity Is Associated with Excessive EZH2 Expression and Enhanced Tumour Invasion in Nasopharyngeal Carcinoma

**DOI:** 10.1371/journal.pone.0068614

**Published:** 2013-07-18

**Authors:** Renqiang Ma, Yi Wei, Xiaoming Huang, Ran Fu, Xi Luo, Xiaolin Zhu, Wenbin Lei, Jugao Fang, Huabin Li, Weiping Wen

**Affiliations:** 1 Allergy and Cancer Center, Otorhinolarygology Hospital, The First Affiliated Hospital of Sun Yat-sen University, Guangzhou, China; 2 Department of Otolaryngology, The Second Affiliated Hospital of Sun Yat-sen University, Guangzhou, China; 3 Department of Otolaryngology, Beijing Tongren Hospital, Capital Medical University, Beijing, China; University of Alabama at Birmingham, United States of America

## Abstract

**Background:**

Enhancer of zeste homolog 2 (EZH2) has been shown to contribute to tumour development and/or progression. However, the signalling pathway underlying the regulation of EZH2 in nasopharyngeal carcinoma (NPC) remains unclear. Since EZH2 contains the putative Glycogen synthase kinase 3 beta (GSK3β) phosphorylation motif ADHWDSKNVSCKNC (591) and may act as a possible substrate of GSK-3β, it is possible that inactivation of GSK3β may lead to excessive EZH2 expression in NPC.

**Method:**

We first examined the expression of EZH2 and phosphorylated GSK3β (p-GSK3β) by immunohistochemical staining in NPC samples. Then, we evaluated the interaction of GSK3β and EZH2 using immunoprecipitation and immune blot. Moreover, we determined the effect of inhibition of GSK3β activity on EZH2 expression and tumor invasiveness in NPC cell lines *in vitro*. Finally, we evaluated the invasive properties of NPC cells after knocking down EZH2 expression with EZH2 siRNA.

**Results:**

We found that expression of EZH2 correlated with phosphorylated GSK3β (p-GSK3β) at Ser 9 (an inactivated form of GSK3β) in human nasopharyngeal carcinoma (NPC) samples. We also provided evidence that GSK3β is able to interact with EZH2 using immunoprecipitation and immune blot. Furthermore, we found that inhibition of GSK3β activity can lead to upregulation of EZH2 in NPC cell lines *in vitro*, with enhanced local invasiveness. By knocking down EZH2 expression with EZH2 siRNA, we found that these invasive properties were EZH2 dependent.

**Conclusion:**

Our findings indicate that GSK3β inactivation may account for EZH2 overexpression and subsequent tumour progression, and this mechanism might be a potential target for NPC therapy.

## Introduction

Nasopharyngeal carcinoma (NPC) is a highly malignant disease with a 5-year overall survival rate of approximately 70% and is one of the most common cancers in southern China. Epidemiological data suggest that NPC formation is a result of the interplay between multiple factors, such as genetic susceptibility, environmental factors, and Epstein–Barr virus (EBV) infection [1]. Although excellent results have been achieved on NPC tumourigenesis, the molecular mechanism underlying NPC pathogenesis and progression has not been fully elucidated [2]. Consequently, the survival rate for NPC has not significantly improved even with the use of radiotherapy, radiochemotherapy or targeted radiotherapy (as adjuvant therapy), and almost 30% to 40% of patients will develop distant metastasis within 4 years [3]. Therefore, it is necessary to elucidate the molecular mechanism(s) underlying local invasion and early distant metastasis of NPC in order to find novel therapeutic targets and develop new modalities of treatment.

Recently, it has been suggested that enhancer of zeste homolog 2 (EZH2) is involved in the pathogenesis of NPC by promoting the transformation of immortalised epithelial cells and enhancing cell proliferation and differentiation [4], [5]. EZH2 is a catalytic subunit of the polycomb-repressive complex 2 (PRC2), which catalyses trimethylation of histone H3 lysine 27 (H3K27me3). PRC2 may recruit other polycomb complexes, DNA methyltransferases, and histone deacetylases, resulting in additional transcriptional repressive marks and chromatin compaction at key developmental loci [6]. Overexpression of EZH2 is a marker of advanced and metastatic disease in many solid tumours, including prostate cancer and NPC [4]–[6]. For example, Tong et al. suggested EZH2 plays a critical role in cell invasion and/or metastasis by repressing E-cadherin during the development and/or progression of NPC [4]. In addition, repression of EZH2 by microRNA-26a is related to the inhibition of NPC cell growth and tumourigenesis [5]. However, the signalling pathway underlying EZH2 regulation in NPC remains unclear.

Glycogen synthase kinase 3 beta (GSK3β) is a serine/threonine protein kinase involved in glycogen metabolism and the Wnt signalling pathway, which plays important roles in embryonic development and tumourigenesis [7]. Active GSK3β is able to phosphorylate substrates, such as β-catenin and Tau, resulting in ubiquitin-mediated degradation. GSK3β activity can be abrogated by direct phosphorylation on the Ser9 residue by phosphatidylinositol 3-kinase (PI3K)/Akt, mitogen-activated protein kinase (MAPK)/p90RSK, or mammalian target of rapamycin/S6K upon a number of extracellular stimuli, such as insulin, epidermal growth factor, or fibroblast growth factor [7]. Wnt signalling inactivates GSK3β through the phosphorylation of the Ser9 residue and prevents it from phosphorylating β-catenin, thus stabilising β-catenin in the cytoplasm [8]. Whereas overexpression of GSK3β can induce apoptosis in several cell types, inactivation of GSK3β has been found to reduce apoptosis. Moreover, increasing evidence shows that GSK3β plays a critical role in linking multiple pathways to regulate cellular apoptosis and tumourigenesis by direct phosphorylation of a broad range of substrates, including translation factor eIF2B, cyclin D1, c-Jun, c-myc, NFAT, cyclic AMP–responsive element binding protein, Tau, and Snail [9].

Since GSK3β demonstrates a preference for pre-phosphorylated (primed) substrates by recognising the consensus sequence S/T-X-X-X-Phospho-S/T [10], [11] and EZH2 contains the putative GSK-3β phosphorylation motif ADHWDSKNVSCKNC (591), EZH2 may be a candidate substrate of GSK3β, and GSK3β inactivation may lead to excessive EZH2 expression in NPC. To test this hypothesis, we examined the expression of EZH2 and p-GSK3β (Ser9) in NPC specimens and investigated the possible regulatory mechanism *in vitro*. Our findings regarding GSK3β-regulated EZH2 expression may be beneficial for understanding the pathogenic mechanism of NPC and improve the prognosis of this disease.

## Materials and Methods

### Ethics statement

The research protocols were approved by the Ethics Committee of the First Affiliated Hospital of Sun Yat-sen University. All NPC and control participants with tissue examination provided their written informed consent to participate in this study.

### Constructs and reagents

The human NPC cell lines CNE-1 and CNE-2 were obtained from the Cancer Center of Sun Yat-sen University. The kinase-dead GSK3β (GSK3β-KD) and constitutively active GSK3β (GSK3β-CA) plasmids were kindly provided by Qingqing Ding from (MD Anderson Cancer Center). Lithium chloride was obtained from Sigma-Aldrich. Antibodies (rabbit anti-human GSK3β, rabbit anti-human p-GSK3β (Ser9) and rabbit anti-human EZH2) were purchased from Cell Signal Technology. Rabbit anti-human GAPDH was obtained from Santa Cruz Biotechnology.

### Patient samples

Primary NPC biopsy specimens (n = 40) and normal biopsies of the nasopharynx (n = 34) were obtained from the First Affiliated Hospital of Sun Yat-sen University. Both tumour and control tissues were histologically confirmed by H&E (hematoxylin and eosin) staining. The demographic characteristics are listed in Table 1.

**Table 1:** Descriptive characteristics of NPC patients and normal controls.

Characteristic	NPC patients	Normal controls
Sex (male:female)		
Male	24	20
Female	16	14
Age (y, mean±SD)	49.3±15.2	33.4±14.5
Histology		
Undifferentiated	40	
Other	0	
Primary tumor stage		
T1	13	
T2	15	
T3	10	
T4	2	
Nodular metastasis		
Yes	27	
No	13	
Distant metastasis		
Yes	1	
No	39	

### Immunohistochemical and immunofluorescent staining

Immunohistochemical staining was performed as previously reported [12]. Briefly, human tissue sections were stained for the expression of phosphorylated GSK3β (Ser9) (1∶200) and EZH2 (1∶200) and detected by streptavidin–biotin–horseradish peroxidase complex formation. Immunoglobulin G was used as a negative control instead of primary antibodies. Two independent observers blind to the diagnoses and clinical data counted the number of positive cells in 5 randomly selected high-power fields (HPFs, 400×), and the numbers were averaged.

### Cell culture, transient transfection and RNA interference

The human NPC cell lines CNE-1 and CNE-2 were cultured in RPMI-1640 supplemented with 10% foetal bovine serum (Hyclone). Transient transfection with GSK3β-CA or KD plasmid (2 μg/mL) was performed with Lipofectamine 2000 reagent (Invitrogen) according to the manufacturer's protocol. In addition, lithium (20 mmol/L) was used to inhibit the activity of GSK3β. For RNA interference, EZH2 siRNA (50 nmol/L) was transfected into NPC cells with Lipofectamine 2000 reagent (Invitrogen) according to the manufacturer's protocol.

### Immunoprecipitation and immune blot analysis

For western blot analysis, whole cell lysates (KeyGEN) were resolved by SDS-PAGE, followed by immunoblotting using antibodies at the following dilutions: anti-p-GSK3β (Ser9) (1∶500), anti-GSK3β (1∶1000), anti-EZH2 (1∶500) and anti-GAPDH (1∶3000). For immunoprecipitation, precleared whole cell lysates were immunoprecipitated with anti-GSK3β (1∶100) insolubilised on protein G Plus/protein A agarose suspension (CalBiochem). Then, immunoprecipitates were loaded on a 10% SDS-PAGE and immunoblotted with different antibodies as described above. Blots were developed using the ECL detection system (BeyotimeBeyoECL Plus).

### Cell migration and invasion assays

Cell migration was measured using the scratch assay as described elsewhere [13]. Briefly, CNE-1 and CNE-2 cells were grown in serum-free medium until 90–100% confluency was reached. After GSK3β-KD or CA plasmid (2 μg/mL) were transfected for 24 h, a 3-mm wound was introduced across the diameter of each plate. The scratch area was measured using ImageJ. The cell covered area was calculated again 48 h after transfection. Cell invasion was detected by transwell invasion assay, which was performed as described elsewhere [14]. Briefly, CNE-1 and CNE-2 cells were grown in serum-free medium until 90–100% confluency was reached. The assay was performed using chambers with an 8 micron pore size polyethylene terephthalate membrane and a thin layer of matrigel basement membrane matrix. After GSK3β-KD or CA plasmid (2 μg/mL) was transfected for 72 h, the cells on the underside of the filter were fixed, stained and counted.

### Statistical analysis

The number of positive cells in tissues was expressed as the median and 25–75^th^ percentile and analysed using a nonparametric Mann-Whitney U-test. The Spearman rank correlation test was used to analyse the correlation among different parameters. The *in vitro* data were expressed as the mean and standard error of the mean (SEM) and analysed using an ANOVA and a two-tailed t-test. A P-value less than 0.05 was considered statistically significant.

## Results

### Correlation between GSK3β inactivation and EZH2 expression in NPC tissues and cell lines

**Figure 1:**
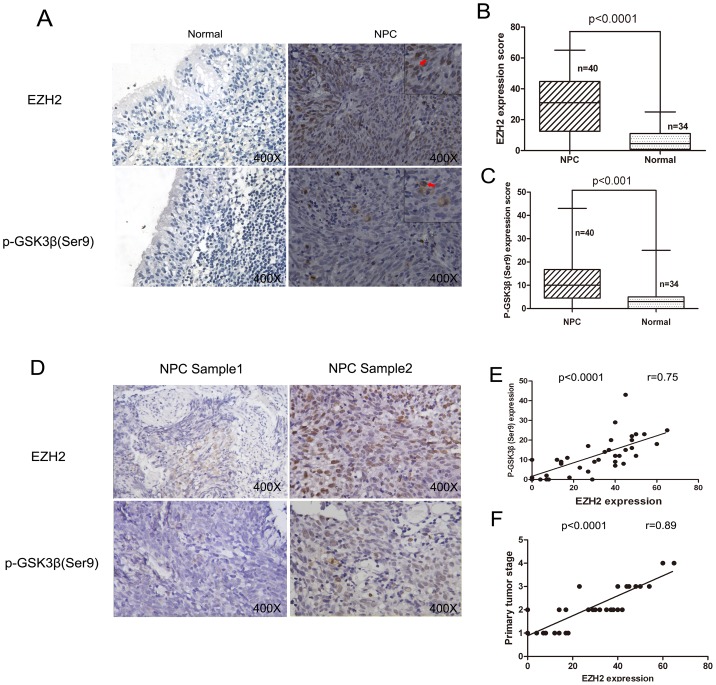
Expression of p-GSK3β (Ser9) and EZH2 in NPC tissues and normal controls. (A) Representative immunohistochemical staining results of EZH2 and p-GSK3β (Ser9) in NPC and control tissues; (B) The mean number of EZH2 positive cells is greater in NPC tissues than in normal controls; (C) The mean number of p-GSK3β positive cells is greater in NPC tissues than in normal controls; (D) Coincident high or low EZH2 and p-GSK3β (Ser9) immunohistochemical staining in 2 representative NPC tissues; (E) Immunoreactivity of EZH2 is positively associated with p-GSK3β (Ser9) immunoreactivity in NPC tissues; (F) Immunoreactivity of EZH2 is positively associated with higher stage of NPC.

Given that EZH2 contains a putative GSK3β phosphorylation motif, we first tested whether there was a correlation between EZH2 expression and GSK3β inactivation in NPC specimens. As shown in Fig 1A, both EZH2 and p-GSK3β (Ser9) protein expression showed specifically nuclear and cytoplasmic distribution. To quantify the expression of EZH2 and p-GSK3β (Ser9), we counted and averaged the number positive cells in 5 randomly selected HPFs. Consequently, we found the mean number of EZH2-positive cells per HPF was 35.4 [14.0, 50.2] and 4.8 [2.0, 13.4] in NPC and control tissues, respectively. Similarly, the mean number of p-GSK3β (Ser9)-positive cells per HPF was 11.2 [7.7, 18.5] and 3.2 [1.0, 5.8], respectively. These results showed that the levels of p-GSK3β (Ser9) and EZH2 immunoreactivity in NPC specimens were significantly higher than those in normal nasopharyngeal tissues (*p*<0.05 for both) (Fig 1B and C). There was a significant association between p-GSK3β (Ser9) and EZH2 immunoreactivity in NPC specimens by Spearman rank correlation (*r* = 0.75, *p*<0.05) (Fig 1D and E). In addition, we evaluated the relationship between EZH2 immunoreactivity and clinical severity of NPC. We found EZH2 immunoreactivity to be positively associated with tumour stage (*r* = 0.89, *p*<0.05) (Fig 1F).

### Evidence for the interaction between GSK3β and EZH2 in vitro

**Figure 2:**
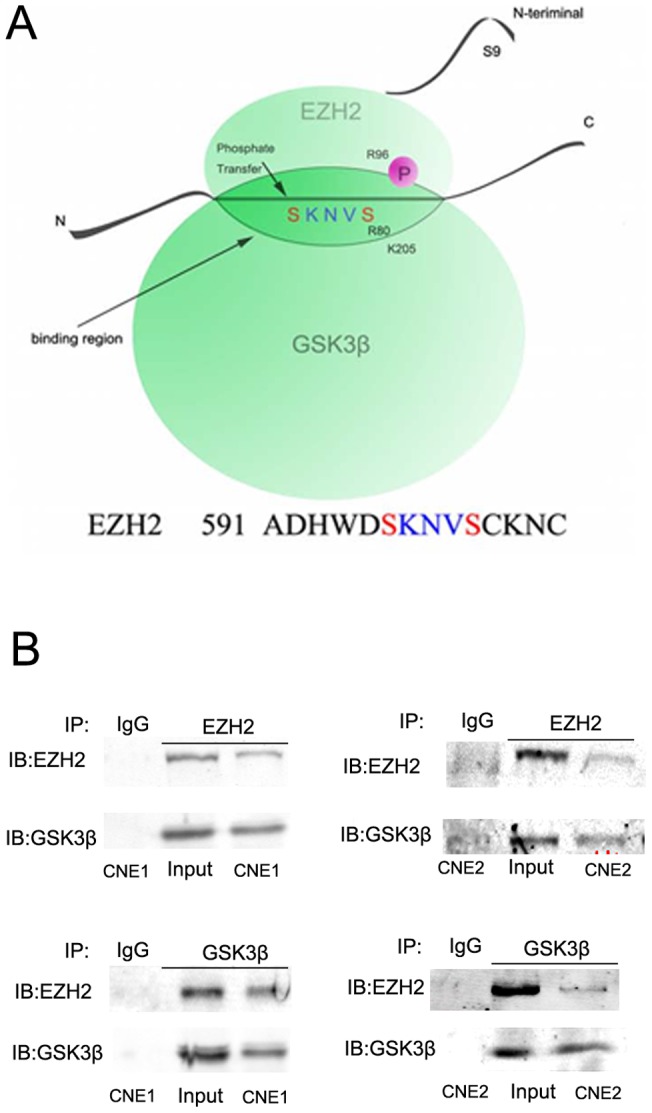
Evidence that GSK3β is able to bind to EZH2 in NPC cell lines. (A) Schematic diagram of the putative GSK3β phosphorylation motif sequence alignment in EZH2; (B) Evidence that GSK3β is able to bind to EZH2, as determined by immunoprecipitation and immune blotting. Lysates from CNE-1 and CNE-2 cells were used for immunoprecipitation. IB: immune blot; IP: immunoprecipitation.

By using molecular structure analysis, we found that EZH2 contains a putative GSK3β phosphorylation motif, ADHWDSKNVSCKNC (591), and thus, EZH2 may be a candidate substrate of GSK3β (Fig 2A). To examine whether GSK3β could interact with EZH2, lysates from CNE-1 and CNE-2 cells were used for GSK3β and EZH2 co-immunoprecipitation. As shown in Fig 2B, the interaction between GSK3β and EZH2 was clearly detected by western blot analysis.

### GSK3β inactivation is associated with EZH2 overproduction *in vitro*

**Figure 3:**
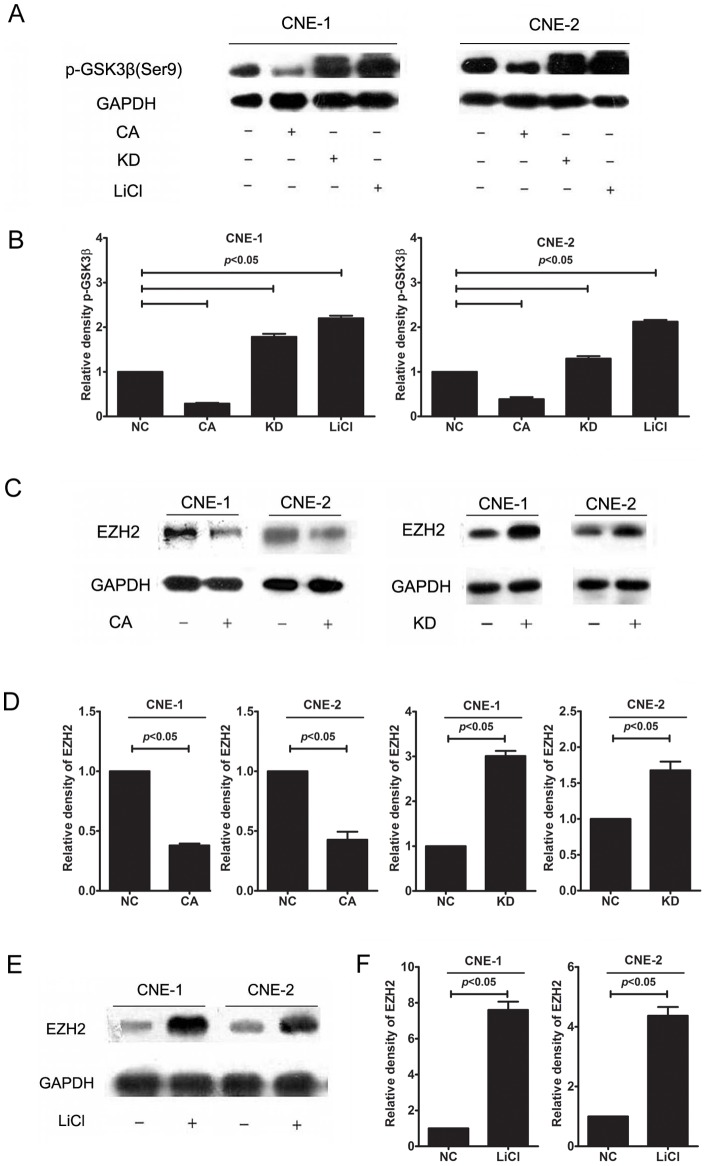
Inactivation of GSK3β upregulated EZH2 production in NPC cells. (A) The transfection efficiency was evaluated by testing the protein level of GSK3β. Representative western blot analysis of p-GSK3β (Ser9) after GSK3β-CA or KD transfection or lithium treatment (20 mmol/L) in NPC cells; (B) GSK3β-CA transfection (2 μg/mL) significantly reduced p-GSK3β (Ser9) production in CNE-1 and CNE-2 cells, whereas GSK3β-KD transfection (2 μg/mL) or lithium treatment (20 mmol/L) significantly increased p-GSK3β (Ser9) production in CNE-1 and CNE-2 cells; (C) Representative western blot analysis of EZH2 after GSK3β-CA or KD transfection in NPC cells; (D) GSK3β-CA transfection (2 μg/mL) significantly reduced EZH2 production in CNE-1 and CNE-2 cells, whereas GSK3β-KD transfection (2 μg/mL) significantly increased EZH2 production in CNE-1 and CNE-2 cells; (E) Representative western blot analysis of EZH2 after lithium treatment (20 mmol/L) in NPC cells; (F) Lithium treatment (20 mmol/L) significantly increased EZH2 production in CNE-1 and CNE-2 cells. The data indicate the means (SEM) of 3 independent experiments. NC: normal control; CA: constitutively active GSK-3β plasmid; KD: kinase-dead GSK-3β plasmid.

To investigate whether GSK3β regulates EZH2 expression, CNE-1 and CNE-2 cells were transfected with GSK3β-CA or KD plasmids, and EZH2 protein expression was examined by immune blot analysis. As illustrated in Fig 3, when CNE-1 and CNE-2 cells were transfected with GSK3β-CA, we observed that both p-GSK3β (Ser9) and EZH2 were significantly downregulated in CNE-1 and CNE-2 cells. However, when GSK3β activity was inhibited after cells were transfected with GSK3β-KD or treated with lithium, both p-GSK3β (Ser9) and EZH2 were significantly upregulated in CNE-1 and CNE-2 cells. These findings provided further evidence that excessive EZH2 expression is associated with the inactivation of GSK3β.

### GSK3β inactivation promoted the stability of EZH2 protein in vitro

**Figure 4:**
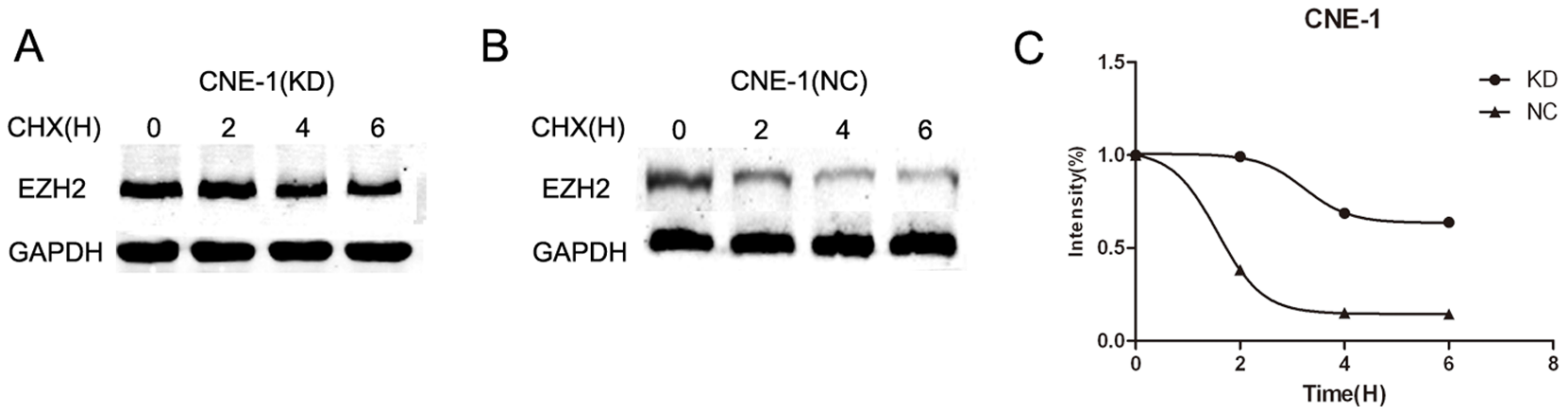
GSK3β inactivation promoted the stability of EZH2 protein *in vitro*. (A, B) Representative western blot analysis of EZH2 after GSK3β-KD (2 μg/mL) or control plasmid transfection in CNE-1 cells; CNE-1 cells were treated with cycloheximide (20 μM) after transfection, and EZH2 protein level in the indicated time point was detected by western blot analysis which containing equal amounts of protein. (C) The half-life of EZH2, as suggested by the relative EZH2 intensity, was significantly longer in GSK3β-KD group than in normal control (*p*<0.05). The data indicate the means (SEM) of 3 independent experiments. KD: kinase-dead GSK3β plasmid; NC, normal control plasmid.

To investigate the molecular mechanism underlying EZH2 expression after inhibition of GSK-3β activity, we examined EZH2 mRNA level in CNE-1 and CNE-2 cells after GSK3β-KD transfection. However, not significant effect of GSK3β inactivation on EZH2 was observed (data not shown). To further investigate whether GSK3β exert a posttranscriptional regulation on EZH2 production, we then examined the stability of EZH2 protein in CNE-1 cells after GSK3β-KD transfection (2 μg/mL) in vitro. As illustrated in Fig 4, when CNE-1 cells were treated with cycloheximide (20 μM) for the indicated times after transfection, we found the half-life of EZH2 protein in GSK3β-KD group was significantly longer than in normal control (p<0.05). Therefore, our finding showed inhibition of GSK3β activity can promote EZH2 overproduction by increasing the stability of EZH2 protein.

### GSK3β inactivation and EZH2 upregulation is associated with enhanced invasive capacity of NPC cell lines in vitro

**Figure 5:**
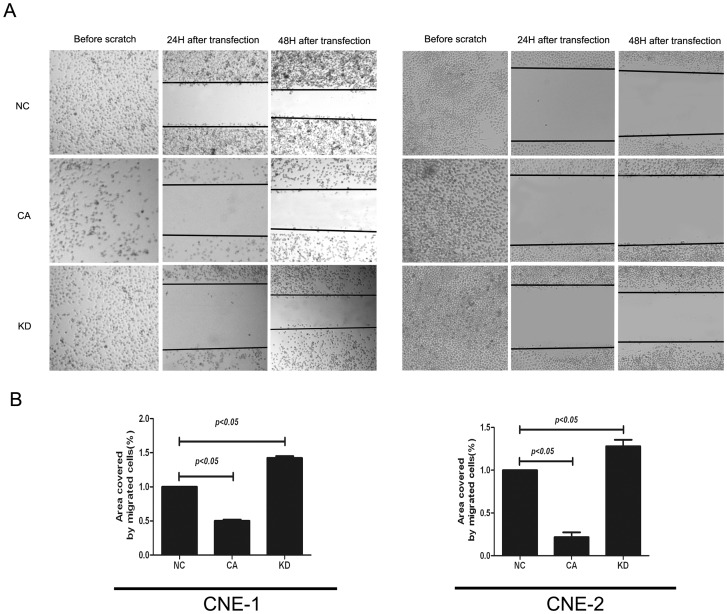
Inhibition of GSK-3β activity enhanced migration of NPC cell lines. (A) Representative images showing the cell covered area on the culture plate containing NPC cells after transfection with GSK3β plasmid. Inhibition of GSK-3β by GSK3β-KD transfection enhanced migration of CNE-1 and CNE-2 cells, whereas activation of GSK-3β by GSK3β-CA transfection suppressed migration of CNE-1 and CNE-2 cells; (B) Quantitative analyses for the cell covered areas showed that the migrated cells in the GSK3β-KD group increased significantly, whereas those in GSK3β-CA group decreased significantly when compared to the control. Migration of NPC cells was evaluated by scratch assay after GSK3β-KD or CA plasmid (2 μg/mL) transfection for 24 or 48 h. The data indicate the means (SEM) of 3 independent experiments. NC: normal control; CA: constitutively active GSK3β plasmid; KD: kinase-dead GSK3β plasmid.

**Figure 6:**
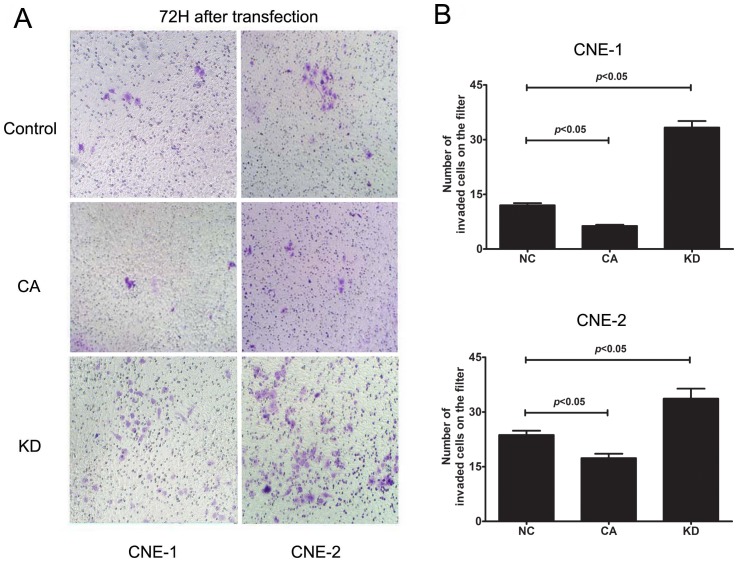
Inhibition of GSK-3β activity enhanced invasion of NPC cells. (A) Representative photos showing the NPC cell density on the filter after transfection with GSK3β plasmid. Inhibition of GSK-3β by GSK3β-KD transfection enhanced invasion of CNE-1 and CNE-2 cells, whereas activation of GSK-3β by GSK3β-CA transfection suppressed invasion of CNE-1 and CNE-2 cells; (B) Quantitative analyses of the number of invaded cells showed that the invaded cells in GSK3β-KD group increased significantly, whereas those in GSK3β-CA group decreased significantly when compared to the control. Invasion of NPC cells was evaluated by transwell assay after transfection with GSK3β-KD or CA plasmid (2 μg/mL) for 72 h. The data indicate the means (SEM) of 3 independent experiments. NC, normal control; CA: constitutively active GSK-3β plasmid; KD: kinase-dead GSK-3β plasmid.

Because EZH2 has been shown to play a critical role in cell invasion and/or metastasis during the tumourigenesis of NPC, we investigated whether GSK3β inactivation and subsequent EZH2 upregulation affected the invasion of NPC cells using the cell scratch assay. As illustrated in Fig 5, after transfection with GSK3β-KD or GSK3β-CA plasmid for 48 h, we found the covered area of migrated cells was significantly smaller in the GSK3β-CA group, where EZH2 was downregulated, but significantly larger in the GSK3β-KD group, where EZH2 was upregulated, when compared to the control group. Moreover, the ability of cells to invade matrigel indicates the invasive capacity of the CNE-1 and CNE-2 cell lines. By transwell invasion assay, we found that the number of invaded cells was significantly less in the GSK3β-CA group and significantly more in the GSK3β-KD group when compared to the control group (Fig 6). Taken together, these findings indicate that GSK3β inactivation enhances the migratory and invasive capacities of NPC cell lines *in vitro*.

**Figure 7:**
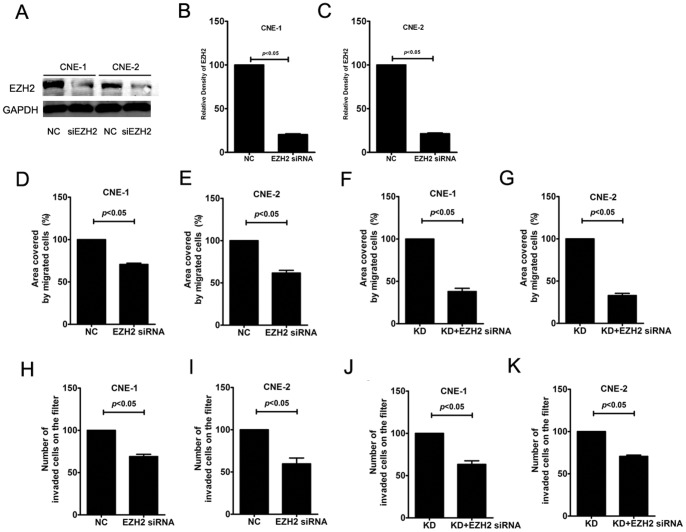
GSK3β-enhanced migration and invasion of NPC cells were abrogated by EZH2 siRNA transfection. (A–C) The siRNA knockdown efficiency was evaluated by testing the protein level of EZH2. EZH2 siRNA transfection significantly reduced EZH2 expression in CNE-1 and CNE-2 cells; (D,E) Inhibition of EZH2 by EZH2 siRNA (50 nmol/L) transfection significantly inhibited migration of CNE-1 and CNE-2; (F,G) EZH2 siRNA (50 nmol/L) transfection significantly inhibited GSK3β-KD-dependent migration of CNE-1 and CNE-2; (H,I) Inhibition of EZH2 by EZH2 siRNA (50 nmol/L) transfection significantly inhibited invasion of CNE-1 and CNE-2; (J,K) EZH2 siRNA transfection significantly inhibited GSK3β-KD-dependent invasion of CNE-1 and CNE-2. Migration and invasion of NPC cells were evaluated after EZH2 siRNA (50 nmol/L) and/or GSK3β-KD plasmid (2 μg/mL) were transfected for 48 or 72 h. The data indicate the means (SEM) of 3 independent experiments. NC: normal control; KD: kinase-dead GSK-3β plasmid.

To further test whether EZH2 was involved in the enhanced invasion of NPC cell lines followed by GSK3β inactivation, we transfected EZH2 siRNA into NPC cells to inhibit EZH2 expression under different conditions. As illustrated in Fig 7, EZH2 siRNA transfection significantly changed the covered area of migrated cells in the scratch assay, as well as the number of invaded cells in the transwell assay. The effects of EZH2 siRNA on the covered area of migrated cells, as well as the number of invaded cells, were especially significant in the GSK3β-KD group. These findings suggest that EZH2 is essential for the enhanced migratory and invasive capacities of NPC cell lines after GSK3β inactivation.

## Discussion

In the present study, we present the preliminary clinical and *in vitro* data suggesting a possible role for GSK3β in the regulation of EZH2 and subsequent progression of NPC. Our findings suggest that an aberrant GSK3β/EZH2 regulatory axis may be critical for initialising the formation of NPC. NPC is known to be a prevalent malignant neoplasm with a distinct epidemiology and geographical distribution. Currently, southern China has the highest risk worldwide, and there are many advanced patients suffering from a poor prognosis. Although the molecular events responsible for the progression of NPC remain to be elucidated, the common mechanism appears to be the aberrant activation of developmental signalling pathways, leading to uncontrolled cell proliferation. By examining the mechanism through which GSK3β regulates excessive EZH2 production, our findings present promising evidence for developing a potential therapeutic target for the future management of NPC.

Gene expression is regulated at a number of different levels, one of which is the accessibility of genes and their controlling elements to the transcriptional machinery. EZH2 can bind the DNA methyltransferases DNMT1, DNMT3A, and DNMT3B, which can result in DNA methylation in certain circumstances [15]. Although several reports in the literature documented overexpression of EZH2 and EZH2-dependent tumourigenesis in human NPC [4], [5], [16], [17], the precise molecular mechanisms leading to EZH2 upregulation remain largely unknown. In agreement with these studies, we observed high EZH2 expression in this group of NPC specimens. EZH2 expression was positively associated with clinical severity, suggesting that EZH2 upregulation can contribute to the local invasion of NPC. Moreover, we found EZH2 expression is significantly related to the inactivation of GSK3β (Ser9) in these NPC specimens. Since GSK3β demonstrates a preference for pre-phosphorylated (primed) substrates by recognising a consensus sequence and EZH2 contains the putative GSK3β phosphorylation motif ADHWDSKNVSCKNC (591), we hypothesised that GSK3β may exert a regulatory effect on EZH2 by site-specific phosphorylation. As we suspected, when GSK3β and EZH2 were co-immunoprecipitated from NPC cell lysates, the interaction between GSK3β and EZH2 was clearly detected by immune blot, indicating GSK3β is able to recognise and bind to EZH2. Due to technical restriction, our working on site-specific phosphorylation of EZH2 is still in progress, we thus are unable to show the evidence of phosphorylation of EZH2 in response to GSK3β in this study. Future data on the specific phosphorylation site of EZH2 by GSK3β transfection is therefore of great interest.

Recently, GSK3β has become an important area of investigation as a key component of the Wnt signalling pathway. Unlike other protein kinase, GSK3β is constitutively active in resting cells and undergoes a rapid and transient inhibition in response to a number of external signals [20]. GSK3β activity is regulated by site-specific phosphorylation as well. Full activity of GSK3β generally requires phosphorylation at tyrosine 216 (T216), and conversely, phosphorylation at serine 9 (Ser9) leads to the inhibition of GSK3β activity [18]. GSK3β also participates in neoplastic transformation and tumour development. The role of GSK3β in tumourigenesis and cancer progression remains controversial; it may function as a “tumour suppressor” for certain types of tumours but promotes growth and development for some others [19]. A variety of signalling pathways may contribute to NPC carcinogenesis. For example, the EBV-encoded latent membrane proteins (LMP1, LMP2A, and LMP2B) have been associated with activation of PI3K/Akt and extracellular signal-regulated kinase (ERK)/MAPK [20], [21], and LMP2A has been shown to activate the protooncogenic Wnt signalling pathway [22]. However, there is scant literature addressing the role of GSK3β in the signalling pathways underlying the carcinogenesis of NPC. In a previous study, we demonstrated that GSK3β inactivation is associated with tumour stage of NPC through regulation of PMS2 [23]. Similarly, Morrison et al. established the significance of GSK3β inactivation in the ubiquitin-mediated degradation and stabilisation of β-catenin production and NPC progression [24].

In this study, although we were unable to identify the specific phosphorylation site of EZH2, but the observed interaction of GSK3β and EZH2 in NPC cells prompted us to further investigate the regulatory effect of GSK3β on EZH2 production *in vitro*. For this reason, we then transfected GSK3β-CA or GSK3β-KD plasmid or used lithium as a specific inhibitor to regulate GSK3β activity in cell lines. Since we observed significant change in half-life of EZH2 protein but not mRNA expression in response to GSK3β transfection, we concluded that GSK3β may exert its effect on EZH2 expression in the protein level. When GSK3β activity was enhanced by transfection with GSK3β-CA, we observed that active GSK-3β production was significantly upregulated and EZH2 production was significantly inhibited in CNE-1 and CNE-2 cells. Moreover, when GSK3β activity was inhibited upon transfection with GSK3β-KD or lithium treatment, both p-GSK3β (Ser9) and EZH2 were significantly upregulated in CNE-1 and CNE-2 cells. This finding suggested there may exist a balance between activated and inactivated form of GSK3β, and the mechanism still need further investigation.

Although we did not exclude other pathways that may be involved in EZH2 overexpression in human NPC tissues, our finding provided the preliminary evidence that EZH2 expression is regulated by GSK3β with phosphorylation on Ser9. EZH2 belongs to the family of polycomb group proteins and plays a master regulatory role in many important cellular processes. There is increasing evidence that overexpression of the EZH2 gene occurs in a variety of human malignancies, and abnormalities of this gene correlate closely with tumour aggressiveness and/or poor patient prognosis [25], [26]. However, the status and function of EZH2 have not yet been clearly documented in NPC. Recently, Lu et al. reported that knockdown of EZH2 induced cell growth inhibition and a G1-phase arrest, and EZH2 overexpression could rescue the growth suppressive effect in NPC cells [5]. Furthermore, Tong et al. demonstrated that expression of EZH2 in NPC cells and nasopharyngeal tissues correlated with clinicopathological features and survival of NPC patients, and the expression levels of EZH2 influenced the invasive capacity of NPC cell lines *in vitro* [4]. In this study, we also found that inactivation of GSK3β and subsequent EZH2 overexpression promoted local invasion of NPC cells. By cell scratch assay, we found migration was significantly enhanced in the GSK3β-CA group with downregulated EZH2 but was significantly impaired in the GSK3β-KD group with upregulated EZH2. Similar effects on cell invasion were observed in the two groups of NPC cells by transwell invasion assays. Taken together, these findings clearly indicate the potential importance of a dysregulated GSK3β/EZH2 axis in the progression of NPC, which might hold significant promise for identifying critical molecular targets and improving NPC therapy.

## Conclusion

In summary, our findings preliminarily indicate that excessive EZH2 production in human NPC tissues may result from inactivation of GSK3β, which was measured by phosphorylated GSK3β on Ser9 residue. Furthermore, we provide evidence that GSK3β is able to bind to EZH2 *in vitro* and that inhibition of GSK3β activity is associated with excessive EZH2 production, which may enhance the local invasion capacity of NPC cells. Therefore, this newly identified mechanism will be helpful to expand the understanding of NPC tumorigenesis and design potential therapeutic strategy for NPC future management.
